# The Vulnerability of Microbial Ecosystems in a Changing Climate: Potential Impact in Shark Bay

**DOI:** 10.3390/life9030071

**Published:** 2019-09-02

**Authors:** Max Reinold, Hon Lun Wong, Fraser I. MacLeod, Julia Meltzer, April Thompson, Brendan P. Burns

**Affiliations:** 1 School of Biotechnology and Biomolecular Sciences, The University of New South Wales, Sydney 2052, Australia; 2 Australian Centre for Astrobiology, The University of New South Wales, Sydney 2052, Australia

**Keywords:** climate change, microorganisms under stress, microbial mats

## Abstract

The potential impact of climate change on eukaryotes, including humans, has been relatively well described. In contrast, the contribution and susceptibility of microorganisms to a changing climate have, until recently, received relatively less attention. In this review, the importance of microorganisms in the climate change discourse is highlighted. Microorganisms are responsible for approximately half of all primary production on earth, support all forms of macroscopic life whether directly or indirectly, and often persist in “extreme” environments where most other life are excluded. In short, microorganisms are the life support system of the biosphere and therefore must be included in decision making regarding climate change. Any effects climate change will have on microorganisms will inevitably impact higher eukaryotes and the activity of microbial communities in turn can contribute to or alleviate the severity of the changing climate. It is of vital importance that unique, fragile, microbial ecosystems are a focus of research efforts so that their resilience to extreme weather events and climate change are thoroughly understood and that conservation efforts can be implemented as a response. One such ecosystem under threat are the evolutionarily significant microbial mats and stromatolites, such as those present in Shark Bay, Western Australia. Climate change models have suggested the duration and severity of extreme weather events in this region will increase, along with rising temperatures, sea levels, and ocean acidification. These changes could upset the delicate balance that fosters the development of microbial mats and stromatolites in Shark Bay. Thus, the challenges facing Shark Bay microbial communities will be presented here as a specific case study.

## 1. Introduction

Microorganisms are recognised as having an essential role in the health and functions of humans, animals, and indeed the global ecosystem itself [1]. Microorganisms and their associated activities are critical for the continued functioning of the biosphere and more broadly the planet and its atmosphere through nutrient cycling [2]. Furthermore, microorganisms were pivotal in reshaping the oceans and atmosphere, such as through the great oxygenation event, facilitating the conditions that permitted the evolution of multicellular organisms [3] and continue to support all higher trophic life forms [4]. Microscopic life is crucial to maintaining a healthy global ecosystem. For instance, marine phytoplankton are responsible for half of photosynthetic CO_2_ fixation and oxygen production globally [5]. Microorganisms are virtually ubiquitous thriving under “extreme” conditions and on essentially all surfaces in contact with the environment whether living or non-living. Interactions of microorganisms with hosts can constitute additional physical barriers against pathogenic infection (by providing an additional “microbial skin”) but even more importantly can augment/provide essential functions with the host [2]. Microbial life is critical for the functioning of both the planet and the life residing on it, and while life could conceivably persist without microorganisms, the quality of life would be drastically reduced [6].

Climate change is a significant issue of the contemporary environmental discourse. The vast majority of public awareness, research, and funding has been given to anthropogenic sources of greenhouse gases and the downstream effects of climate change on plants and animals [7]. This has resulted in a relatively comprehensive understanding of losses of macro-species, communities and habitats [8]. In contrast, microorganisms have largely been omitted from the discussion revolving around the changing climate, both in their contribution to it (through the absorption and emission of greenhouse gases) and the potential impact of climate change on microbial ecosystems [7]. Moving forward, it is critical that microbial community composition is included in ecosystem modelling so that more accurate projections of climate change effects can be disseminated, leading to heightened public awareness and more informed policy-making [2,7]. This increased awareness is evident by a “call to arms” discussed in recent reviews [2,4], with many scientists urging for the improvement of societal microbiology literacy for this reason.

## 2. Microorganisms and Changing Climate

The longevity of microbial life on earth stretching back to approximately 3.85 billion years suggests that microorganisms are highly resilient and resistant to environmental stressors and change [9]. This concept is supported by the well-documented ability of microorganisms to adapt to a range of environmental threats, including but not limited to, the development of antibiotic resistance (response to antimicrobial agents), the formation of spores (response to starvation), and the activation of motility (to facilitate movement to more beneficial environments) [10,11,12]. The effective response of bacteria to environmental stresses is facilitated by their genetic plasticity which manifests in the form of rapid mutations and horizontal gene transfer (HGT). 

Until recently it was widely thought that microbial communities were resistant and/or resilient to invasion, this belief was propagated by the concept of “colonisation resistance” of the human microbiome [10,11,12,13,14,15,16]. This broad concept was further supported by studies describing disturbances in soil, aquatic, engineered, and human-associated ecosystems merely pushing community taxonomic profiles towards a new stable state while maintaining relatively the same functional potential [17,18,19,20,21]. Even in such cases, however, the perturbation history as well as the time taken for a community to reach this new state of equilibrium is an important factor [19]. In recent years, increasing evidence has suggested that microbial communities may not be as resistant and resilient to disturbances as previously thought, with some ecosystems being unable to recover entirely within several years following a stress event [18]. Several studies highlighted that major compositional changes in gut communities can impact functional potential [22,23] and lead to long-term changes, even if conditions before the perturbation are re-established [20,24]. Resilience refers to the capacity of a community to recover from perturbations (e.g., extreme weather events, rising temperatures). A comprehensive study examined a number of investigations into microbial responses to disturbances, finding that 82% reported sensitivity to disturbance [18]. This number could be skewed as experiments exhibiting no community change when exposed to a disturbance may not be as widely disseminated in the literature [18]. In spite of this, the general conclusion can be drawn that microbial ecosystems and communities are more vulnerable to disturbances and stress events than previously thought [18]. 

Generally speaking, the two major categories of disturbance response for microbial ecosystems are functional and compositional alterations. The two major disturbance types are pulse (short term disturbances, e.g., extreme weather events), and press (longer term disturbances, e.g., increasing greenhouse gases in the atmosphere) [25]. The responses of microbial communities to disturbances are dependent on a number of complex interrelated factors including the type, number, length, and severity of disturbance, the functional and compositional parameters of the microbial ecosystem, and the degrees of functional redundancy [18]. A community with a high level of functional redundancy often enjoys improved stability in the face of environmental and anthropogenic stresses and vice versa [26]. 

While it is true that single-celled organisms have evolved a broad array of stress response mechanisms to increase tolerance to chemical or physical stresses, in the context of an ecosystem community-level responses are more complex. Physiological responses of microorganisms that assist their survival in the face of environmental stress factors can also have the secondary effect of altering ecosystem function and composition [27]. This can potentially manifest in a variety of ways. For instance, since pre-industrial times oceanic pH has reduced by ~0.1 pH units with more severe acidifications of 0.3–0.4 pH units projected before the 22nd century [28,29]. The exact scope of how marine life will respond to these drastic changes in pH is unknown, however acidification causes some microorganisms to alter gene expression to promote cell maintenance rather than growth [30]. Increasing temperatures, caused by elevated atmospheric gases impact biological processes, and decrease water density which in turn decreases stratification, affecting the circulation of nutrients and organisms in marine environments [4]. Another unknown factor is the contribution of seafloor methanogens and methanotrophs producing and consuming CH_4_, to atmospheric concentrations of this greenhouse gas [31]. Microorganisms in soil regulate the approximately 2000 billion tonnes of organic carbon stored in soils, determining how much is stored and how much is released back to the atmosphere [32]. The balance achieved by plants fixing CO_2_ through photosynthesis and the release of CO_2_ via autotrophic and heterotrophic respiration by plants and microorganisms respectively is influenced by temperature. Global warming is predicted to accelerate the release of CO_2_ into the atmosphere [33]. It is clear that the effects of climate change on microorganisms and their respective ecosystems are wide-ranging.

Even if a microbial ecosystem has a limited response to a stress event, the physiological changes that occur can alter the flow of nutrients. As a response to drought conditions microorganisms synthesize osmolytes using carbon (C), nitrogen (N), hydrogen (H), and oxygen (O), to maintain fluid balance and cell volume. In grassland ecosystems, a single drought episode can result in the consumption of up to 5% of annual net carbon production [27]. Arctic tundra soils immobilize nitrogen (N) during the warmer season and mineralize N during the winter as a means of acclimating to freezing conditions [27]. Thus, drought and freezing conditions alter the allocation and fate of C and N [27]. Another study assessed the response of soil microbial communities to drying and rewetting stress in an attempt to reflect climate model projections of increased duration and frequency of droughts and large precipitation events, respectively [34]. Soil samples were acquired from a long-term field manipulation (Rainfall Manipulation Plot Study) where rainfall conditions were experimentally varied. After obtaining the soil samples they were subjected to a series of drying–rewetting pulses under controlled conditions [34]. After the drying and rewetting stresses, respiration, fungal:bacterial ratios, bacterial community composition, and microbial biomass were measured [34]. In short, the results obtained indicated that environmental history affect biogeochemical processes in the present. Soils accustomed to higher levels of rainfall were more resistant to the experimental pulses. The community composition of bacteria was altered after drying–rewetting treatment and the degree of this alteration depended on their previous rainfall conditions [34]. Recently a new two-step model for describing community resilience was outlined as a potential framework for future research [35]. The model was first harnessed to characterise the response of an extremophile community inhabiting halite (salt rocks) in the Atacama Desert to a significant rainfall event [35]. Shotgun metagenomic sequencing of microbiomes was conducted over a 4-year longitudinal study with observed changes that occurred due to the rainfall event being recapitulated into two modes of community shift. *Type 1* shift describes the rapid initial response whereby the community sat in an unstable intermediate state, within which protein adaptations to increased water availability occurred as a consequence of niche re-colonization. *Type 2* adjustment describes the communities’ return to its former functional potential by long-term adjustments in the abundances of newly acquired taxa [35]. 

There is an immense amount of data that have been reported on the high sensitivity of microbial communities to natural and anthropogenic stressors, however these varied studies are difficult to compare due to the lack of a systematic database. However, a global collaborative initiative, the Microbiome Stress Project [36], is attempting to address this. Furthermore, the resilience of natural communities following environmental disasters and stress events, continue to be largely unexplored, with most studies relying on manipulative experiments under controlled conditions due to the multitude of compounding environmental factors in a natural setting [37,38].

Microbial ecology, population biology, and process ecology need to be more comprehensively understood and integrated into ecosystem ecology to generate a complete picture of stress events on ecosystems [4,27]. The resilience and resistance of each microbial ecosystem to different sources of disturbance will vary drastically, and each ecosystems response to different stress events may alter the balance of the global biosphere. Thus, each ecosystem needs to be observed and studied specifically for each source of stress and adequate attempts made to incorporate this into a global context. An example of a particular ecosystem that is under the threat of climate change is microbial mats and stromatolites, such as those found in Shark Bay, Western Australia. 

## 3. Microbial Mats and Stromatolites

### 3.1. Description and Characteristics

Microbial mats are layered, laminar, organo-sedimentary, microbial ecosystems, composed of microorganisms usually embedded in an organic biofilm matrix in which extracellular polymeric substances (EPS) provide both functional and structural integrity by forming a cohesive structure [39]. EPS are secreted by mat microorganisms and are primarily composed of exopolysaccharides, proteins, and extracellular DNA (eDNA), as well as smaller amounts of lipids and humic substances [40]. The matrix surrounding microbial mat microorganisms also contains minerals such as silicates and carbonates [41,42,43]. Microbial mats predominantly develop at the interface of sediment substrate and water in a range of diverse aquatic habitats such as hot springs, hypersaline ponds, and intertidal coastal zones [44,45]. They usually form in shallow water or on moist surfaces due to the trapping, binding, and precipitation activity of resident microbial communities. The primary source of nutrition and energy for most mats is acquired through photosynthesis conducted by cyanobacteria [46]. Photosynthesis harnesses sunlight to fix atmospheric carbon dioxide (CO_2_) to organic carbon that can be used as energy ((CH_2_O)_n_) with oxygen produced as a bi-product [47]. A significant proportion of cyanobacteria as well as other mat bacteria and archaea can also fix atmospheric nitrogen (N_2_), highlighting the complexity of microbial mat ecosystems [47,48]. In addition, other processes such as denitrification, metal reduction, sulfate reduction, and methanogenesis are also critical to mat survival [49]. The onset of “omics” technologies has enabled studies into the biodiversity of microbial mats at high resolution and depth and has been utilised to highlight the compositional diversity of these microbial ecosystems. For example, one study of mats in Guerrero Negro delineated 750 different species [46], and studies in other locations have revealed that microbial mats house a multitude of microorganisms belonging to different taxonomic and functional groups [50,51,52,53]. Microbial mats are complex microbial ecosystems that are propped up by multidimensional interactions at a fine millimetre scale [52]. The distribution of microorganisms throughout microbial mats are putatively determined by availability of oxygen and other nutrients, as well as light.

As briefly covered above, within microbial mats, nutrients such as carbon, oxygen, nitrogen and sulfur are metabolised and cycled, and microbial mats are thus major contributors to biogeochemical cycling. Often these nutrients (as well as a matrix of exopolysaccharides) encapsulate the microorganisms residing in the microbial mat, allowing for more efficient cycling of resources and energy, improving the functionality of the microbial community [54]. Given the close proximity of microorganisms residing in microbial mats, biochemical processes and biogeochemical cycles are coupled such that products produced by the metabolic activity of one group of microorganisms are available to be used almost immediately by other groups of microorganisms in the mat [55]. Microbial mats can sometimes mediate the formation of microbialites (microbially induced mineral precipitations) [53], such as stromatolites.

### 3.2. Evolutionary Significance

Fossil stromatolites are the oldest form of life for which there is a reliable fossil record [56]. They are touted as the first ecosystems on earth and also comprise a large percentage of the fossil record of life stretching back to the Archaean era (oldest potentially from ~3.7 Ga) [43,45,57]. Extant stromatolites and microbial mats are analogues of these ancient fossils, exhibiting morphology similar to their fossilized counterparts. As a result, stromatolites are often referred to as “the oldest living fossils” and are therefore capable of providing key information into early ecosystems on earth [42,52,58]. While microbial mats and stromatolites have existed on earth since 3.7 Ga [57], their distribution exploded globally during the Proterozoic era (2.5–0.57 Ga) [59]. 

Around 1.25 Ga, stromatolites peaked in abundance and were common across the globe, however, the abundance of stromatolites subsequently decreased after this period [60]. This is thought to have occurred due to grazing by eukaryotic and multicellular organisms. Metazoan burrowing and grazing have been suggested to inhibit the growth and development of microbial mats and, by extension, stromatolites [61]. Indeed, improved burrowing capabilities of multicellular organisms living in shallow seas have been thought to drive mats off these benthic zones [41]. Thus, stromatolites exist in aquatic habitats where metazoan grazing is mostly inhibited due to environmental conditions such as in hypersaline lakes and hydrothermal vents [61]. Microbial mats flourish in extreme environments [45] but are found in a wide variety of habitats such as coastal and shelf settings and in less complex forms on rocks, and within soil. Elucidating the role ancient microbial mats played in the development and maintenance of the earth’s geochemical environment is difficult because microbial mat fossils are generally not well preserved. However, lithified mats formed through carbonate precipitation can improve the preservation of fossils [62]. What has been discerned from the fossil record of stromatolites seems to strongly suggest that microbial mat and thus stromatolite communities were highly resistant and resilient to environmental changes [59]. As mentioned previously however (e.g., concept of “colonization resistance”), it is important not to assume the resistance and/or resilience of specific microbial ecosystems based on their longevity, and thus focused study is needed to determine the potential impact—or lack thereof—that climate change could have on microbial mats and stromatolites.

Microbial mats are generally believed to be the first photosynthetic communities (potentially the first microbial community) that developed on earth and have been described as the forests of the past [56]. Indeed, ancient microbial mats are thought to have been responsible for the Great Oxygenation Event (GOE). The GOE began in the early Paleoproterozoic era between 2460–2426 million years ago and describes the first notable build-up of atmospheric oxygen [63]. The evolution of oxygenic photosynthesis is theorised to have increased global biological productivity between 100–1000 times [64], with much of this activity putatively occurring in microbial mats. It is widely believed that the evolution of oxygenic photosynthesis by the ancestors of extant cyanobacteria fuelled the GOE, although this notion has recently been challenged [56]. Microbial mat communities are phylogenetically and metabolically diverse, which challenges the overly simplistic idea that phototrophic mats were initially built by anoxygenic photosynthetic bacteria and later by cyanobacteria [56]. Microbial mats contributed to ocean oxygenation through the metabolic activity of photosynthesis. The surrounding water became saturated until oxygen was released into the air, allowing for the evolution of oxygen dependant organisms such as plants, animals and humans. Production of O_2_, H_2_, and CH_4_ by microbial mats also emphasises their importance as a subject of study for the production of reduced gases on the early earth [65]. 

Microbial mats and stromatolites are thus critical for providing a window into the evolution and development of life on earth. Their importance in this regard should not be understated, a fact that was reinforced recently with the discovery of potential novel organisms under the archaeal Asgard group [58], a proposed superphylum that is suggested to be the closest relatives of eukaryotes [66].

## 4. Shark Bay, Western Australia

**Figure 1:**
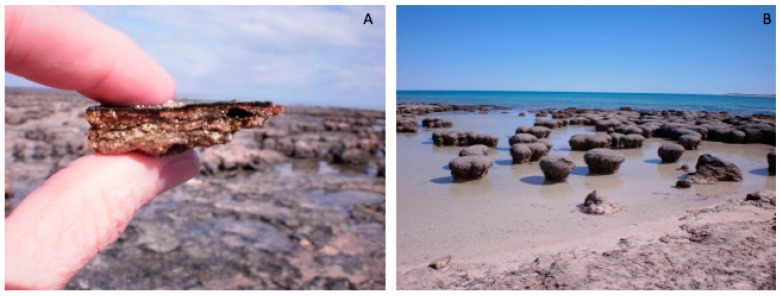
Images of (**A**) microbial mats and (**B**) stromatolites in Shark Bay, Western Australia.

Hamelin Pool in Shark Bay, Western Australia houses one of the most expansive and diverse extant microbial mat and stromatolite systems (Figure 1) [50,67]. It is an ideal site for study into the effects of environmental stresses on microbial mats, specifically those caused by anthropogenic climate change. It is critical that the impacts of stress events on microbial mats and stromatolites are thoroughly understood so that effective responses can be initiated, especially given the importance these communities of microorganisms have in providing a window into the history of life on earth [68]. Furthermore, the harsh conditions that many microbial mats form under (e.g., hypersalinity or high temperature) impart on them potential specific stress survival mechanisms that may have biotechnological applications [45]. For example, antimicrobial compounds and inhibitors of quorum sensing have been described in cyanobacterial mats near hot springs [69]. 

The hypersaline conditions in Hamelin Pool, Shark Bay provide optimal conditions for the formation of microbial mats and stromatolites, potentially via minimizing metazoan grazing and competition for growth factors. The water in Hamelin pool is twice as saline as normal seawater, with salinity over > 60 practical salinity unit (PSU) and they are also subjected to high UV radiation. 

Shark Bay was listed as a UNESCO World Heritage Site in 1991 and is one of only 21 sites worldwide that fulfils all four natural criteria for outstanding universal value (OUV) [70]. These four criteria are: (1) contains superlative natural phenomena or areas of exceptional natural beauty and aesthetic importance; (2) being outstanding examples representing major stages of earth’s history, including the record of life, significant on-going geological processes in the development of landforms, or significant geomorphic or physiographic features; (3) being outstanding examples representing significant on-going ecological and biological processes in the evolution and development of terrestrial, fresh water, coastal and marine ecosystems and communities of plants and animals; and (4) contains the most important and significant natural habitats for in-situ conservation of biological diversity, including those containing threatened species of outstanding universal value from the point of view of science or conservation.

The microbial mats found in Hamelin Pool in Shark Bay contribute heavily to the outstanding universal value (OUV) of the region. Indeed, in the statement of OUV for Shark Bay mats and stromatolites were noted as the most recognisable features of the region [70]. Extensive seagrass beds covering more than 4000 km^2^ are also found in Shark Bay which provide food, shelter and nursery areas for dugongs, bottlenose dolphins, and a host of other marine animals [70]. The bay is also a natural habitat to 12 threatened reptile species, and five endangered mammal species [70]. There is also a thriving tourist industry in the Bay and a multitude of fisheries. Finally, there are five aboriginal language groups in Shark Bay, illustrating the significant cultural importance of conservation in the region.

### 4.1. Functional Properties of Shark Bay Microbial Mats and Stromatolites

Recent advances in metagenomics have facilitated an unprecedented view of the taxonomic and functional diversity of the microbial mats in Shark Bay. Using 16S rDNA analyses, *Actinobacteria*, *Bacteriodetes*, *Chloroflexi*, *Cyanobacteria*, *Gemmatimonadetes*, *Planctomycetes*, *Alphaproteobacteria*, *Gammaproteobacteria*, *Deltaproteobacteria*, *Verrucomicrobia*, *Halobacteriales*, *Euryarchaea*, *Thaumarchaea*, *Parvarchaeota*, *Proteobacteria*, *Acidobacteria*, and a host of other phyla have been described [50,51,52]. Broadly speaking, the top layers of microbial mats are usually dominated by cyanobacteria and more restricted numbers of *Alphaproteobacteria*, and *Bacteriodetes*. In deeper layers of mats where oxygen is severely limited, an abundance of *Chloroflexi* and *Deltaproteobacteria* was observed [51].

Sequencing of Shark Bay stromatolite and microbial mat communities have also described a range of metabolic pathways employed in the cycling of carbon, nitrogen, sulfur and phosphorous [58]. The most highly represented genes were associated with methanogenesis, sulfate assimilation, phosphate transport, Wood–Ljungdahl pathways, and copper efflux [58]. The main mechanism proposed to fix atmospheric carbon into organic carbon was through the Wood–Ljungdahl pathway. Genes linked with energy production/metabolisms were found primarily in the photo-oxic zone, indicating that the majority of energy utilised in mat communities is likely generated at the surface layer [58]. However, there was also a relative abundance of anaerobic pathways in surface layers, possibly suggesting the existence of putative surface suboxic micro-niches in the mats of Shark Bay. 

As described earlier, the hypersalinity of Hamelin pool fosters optimal conditions for microbial mat persistence and survival. However extreme weather events and alterations to established environmental norms associated with climate change threaten to upset the delicate balance that supports the presence of microbial mats and stromatolites in Shark Bay. 

### 4.2. Shark Bay Microbial Mats and Stromatolites under Threat

The hypersalinity observed in Hamelin Pool is facilitated by two main factors. The first being the Faure Sill, a massive elevated seagrass bank that restricts tidal flow to Hamelin pool, leaving the area relatively isolated from oceanic waters [70]. The second factor is that water loss due to evaporation exceeds freshwater input [70,71]. 

A marine heatwave in November 2010 emphasised the fragility of the ecosystems in Shark Bay. During the significant stress event, 36% of the seagrass meadows in the bay were lost [72], and two years on, there was minimal recovery and a decrease in below ground mass [70]. The health status of the largely herbivorous green turtles (*Chelonia mydas)* also declined [73]. This is especially critical in the case of the microbial mats as the seagrass meadows ensure maintenance of the hypersalinity observed in Shark Bay. The marine heatwave also impacted birth rates in dolphins and negatively impacted blue swimmer crab, oyster, and brown tiger prawn fisheries. Tourism visitation also fell drastically below expectations for the year. 

As climate change impact worsen, marine heatwaves have increased in frequency [74] leaving ocean ecosystems highly vulnerable due to their high levels of biodiversity [75]. Sea-level rise could also cause the flooding of Faure Sill, increasing tidal flow into Hamelin Pool and therefore decreasing salinity, which would once again impact mats and stromatolites, generating more favourable conditions for species that could outcompete these systems [70]. Drought and precipitation events are also projected to increase in duration and severity [70]. Periods of heavy and/or prolonged rainfall would decrease salinity by diluting water while periods of drought risk the desiccation of mats and dry out regions near shore, which may have a detrimental impact on stromatolite and mat communities here that appear to be adapted to higher salinity. Ocean acidification, air temperature and water temperature changes also alter the status quo that the extant microbial mats of Hamelin pool have adapted to [70]. Storms are also expected to increase in both intensity and frequency and may destroy mats, increasing turbidity, and potentially smothering the stromatolite building microbial mats. 

In 2015, cyclone Olwyn (category 3 tropical cyclone) made landfall on the western coast of Australia and hit Shark Bay. The impact of such extreme weather events on the mat ecosystems residing in Shark Bay are poorly understood, and information on any observed impact would be beneficial to both management and conservation. The impact of a 1999 weather system (Hurricane Floyd) on cyanobacterial mats in the Bahamas indicated only minor changes to microbial community composition pre and post hurricane as a consequence of “freshening” by rainwater input being observed [71]. Although lake water salinities varied drastically across the study period, the accretion rates of mats increased post-hurricane. Furthermore, evidence suggested that the hurricane stimulated photosynthesis and N_2_ fixation, indicating that the storm events improved the metabolic activity of these specific mats [71]. This kind of experimental design is known as the “Before-After” only model and is a suboptimal design for environmental effects monitoring [76]. The optimal method, known as the Before-After–Control–Impact (BACI), where the control is a similar environmental system that did not undergo the same impact/stress event, is a superior method. Unfortunately, in the case of mat systems, given the variability of stress events, reliable control systems are lacking [76]. Although before–after only studies have limitations, they are still valuable tools for generating a broad picture of an environmental systems response to stress [76], especially as the climate change impact described above along with a host of other changes to the climate are predicted to increase in frequency and intensity.

## 5. Conclusions

Modern microbial mats and stromatolites represent an invaluable area of interest for research on early life on earth, biogeochemical cycling, and the possibility of life elsewhere. The impact of climate change and its associated environmental events on these systems is poorly understood, however, and the fragile balance that allows the maintenance of many of these systems are under threat due to rising sea levels, elevated sea and air temperatures, and a host of other climate change associated effects. To ensure that these evolutionarily significant formations are conserved for future generations, it is essential that their resilience and/or susceptibility in the face of environmental change are thoroughly understood before any irreversible ecosystem tipping points are reached.
